# Impact of Frequency of Multi-Vitamin/Multi-Mineral Supplement Intake on Nutritional Adequacy and Nutrient Deficiencies in U.S. Adults

**DOI:** 10.3390/nu9080849

**Published:** 2017-08-09

**Authors:** Jeffrey B. Blumberg, Balz B. Frei, Victor L. Fulgoni, Connie M. Weaver, Steven H. Zeisel

**Affiliations:** 1 Antioxidants Research Laboratory, Jean Mayer USDA Human Nutrition Research Center on Aging and Friedman School of Nutrition Science and Policy, Tufts University, Boston, MA 02111, USA; 2 Linus Pauling Institute and Department of Biochemistry & Biophysics, Oregon State University, Corvallis, OR 97331, USA; balz.frei@oregonstate.edu; 3 Nutrition Impact, LLC, Battle Creek, MI 49014, USA; VIC3RD@aol.com; 4 Department of Nutrition Science, Purdue University, West Lafayette, IN 47907, USA; weavercm@purdue.edu; 5 Nutrition Research Institute, Department of Nutrition, University of North Carolina, Kannapolis, NC 28081, USA; steven_zeisel@unc.edu

**Keywords:** dietary supplements, nutritional supplements, multi-vitamin/multi-mineral, NHANES, vitamins, micronutrient, nutritional adequacy, nutrient deficiencies

## Abstract

Although >50% of U.S. adults use dietary supplements, little information is available on the impact of supplement use frequency on nutrient intakes and deficiencies. Based on nationally representative data in 10,698 adults from the National Health and Nutrition Examination Surveys (NHANES) 2009 to 2012, assessments were made of intakes from food alone versus food plus multi-vitamin/multi-mineral supplements (MVMS) of 17 nutrients with an Estimated Average Requirement (EAR) and a Tolerable Upper Intake Level (UL), and of the status of five nutrients with recognized biomarkers of deficiency. Compared to food alone, MVMS use at any frequency was associated with a lower prevalence of inadequacy (*p* < 0.01) for 15/17 nutrients examined and an increased prevalence of intakes >UL for 7 nutrients, but the latter was ≤4% for any nutrient. Except for calcium, magnesium, and vitamin D, most frequent MVMS use (≥21 days/30 days) virtually eliminated inadequacies of the nutrients examined, and was associated with significantly lower odds ratios of deficiency for the examined nutrient biomarkers except for iron. In conclusion, among U.S. adults, MVMS use is associated with decreased micronutrient inadequacies, intakes slightly exceeding the UL for a few nutrients, and a lower risk of nutrient deficiencies.

## 1. Introduction

Adequate intake of micronutrients (vitamins and nutritionally-essential minerals) is required for nearly all metabolic, developmental, and growth processes, and for good health across the lifespan. Despite repeated recommendations from the Dietary Guidelines Advisory Committee [1], many Americans have inadequate intakes of several essential nutrients. The recent 2015–2020 Dietary Guidelines for Americans (DGA) [2] identified vitamins A, D, E, and C, and choline, calcium, magnesium, iron (for certain age/gender groups), potassium, and fiber as “underconsumed nutrients”; of these, vitamin D, calcium, iron, potassium, and fiber are under-consumed to the extent that may lead to adverse health outcomes and, as such, were designated as “nutrients of public health concern”. The DGA [2] recommend consuming nutrient-dense foods as part of a healthy eating pattern and, in some cases, fortified foods and dietary supplements, especially for certain population groups.

Dietary supplement consumption has increased over time in the United States [3] and currently appears to have stabilized to about 50% of adults, of whom more than two-thirds use multi-vitamin/multi-mineral supplements (MVMS) [4,5,6]. A recent study [6] reported use of MVMS (defined as ≥10 vitamins and/or minerals at any level) has declined from 37 to 31% from 1999 to 2012 in U.S. adults 20 years and older. In addition to MVMS, single nutrient supplements, especially of vitamins C and E, and calcium and iron, are also commonly used by Americans [7,8]. Supplements are mostly perceived as a favorable health and lifestyle choice, and the key motivators for consumers are maintenance or improvement in overall health as well as specific health benefits rather than filling nutritional gaps [9,10]. Interestingly, there is no standardized definition of MVMS and a wide range of definitions encompassing a wide range of products of different compositions and characteristics are available in the marketplace [6,11]. This heterogeneity poses a significant challenge to studying the effects of MVMS, especially in a nonclinical setting.

The consumption of dietary supplements has been shown to increase overall nutrient intake and decrease the prevalence of nutrient inadequacies [12]. Based on a definition of MVMS as providing at least 100% of the recommended dietary allowance (RDA) or adequate intake (AI) for at least 9 vitamins and minerals, and using data from the National Health and Nutrition Examination Survey (NHANES) 2007–2010, MVMS were reported to contribute to a greater number of individuals meeting their recommended intakes of almost all micronutrients [5]. MVMS users in that study were defined as those who reported using MVMS any time during the last 30-day period. However, there is a great deal of diversity in the frequency of MVMS usage. A recent survey from Korea reported that only 60% of supplement users consume them every day [13]. To date, no studies have been reported examining the impact of frequency of supplement use on nutrient intakes in the U.S. population.

The U.S. Centers for Disease Control and Prevention (CDC) Second National Report on Biochemical Indicators of Diet and Nutrition in the U.S. Population reported that in 2003–2006 less than 10% of individuals had nutritional deficiencies based on measured nutrient biomarkers but, for most nutrition indicators, deficiencies varied by age, gender, or race/ethnicity and were as high as nearly one third of certain population groups [14]. Few studies have reported results of MVMS consumption on biomarkers of nutrient status. The primary objective of this cross-sectional study was to investigate the effect of frequency of MVMS consumption on nutrient intake, prevalence of inadequacies, and prevalence of deficiencies using a large, nationally representative NHANES data set.

## 2. Materials and Methods

### 2.1. Study Population

This study analyzed data from NHANES, a yearly assessment by the National Center for Health Statistics (NCHS) of the health and nutrition status of a nationally-representative sample of noninstitutionalized U.S. civilians. The data from NHANES 2009–2010 and 2011–2012 surveys were combined for all analyses except where noted. The combined sample included 10,698 adults (5349 males and 5349 females) age 19 years and older, excluding pregnant and/or lactating females and those with incomplete or unreliable data as judged by the USDA Food Surveys Research Group staff. All participants or proxies provided written informed consent and the Research Ethics Review Board at the NCHS approved the survey protocol [15].

### 2.2. Micronutrient Intake from Food

Dietary intake data from two reliable 24-h recall dietary interviews using United States Department of Agriculture’s (USDA) automated multiple-pass method (AMPM) were used [15]. The Food and Nutrient Database for Dietary Studies (FNDDS) 2009–2010 and 2011–2012 [16,17] were used in conjunction with USDA National Nutrient Database for Standard Reference (SR) releases 24 and 26 [18], respectively, to determine the nutrient content of food consumed by subjects who participated in NHANES 2009–2010 and 2011–2012. 

### 2.3. Definition of MVMS and Micronutrient Intake from MVMS

MVMS were defined as dietary supplements providing at least 100% of the RDA or AI for at least 9 vitamins and minerals with defined dietary reference intake (DRI) values for which NHANES 2009–2010 and 2011–2012 collected intake data. Specific EAR and AI values [19,20] for each adult age/gender group were used. In NHANES, a dietary supplement questionnaire assessing the usage of vitamins, minerals, botanicals, and other dietary supplements over the past 30 days was administered as part of the NHANES household interview [21]. The consumption frequency (i.e., number of days the product was taken in the past 30 days), the duration (i.e., how long the product was taken), and the amount usually consumed on days the supplement was taken in the past 30 days were recorded for each supplement. The complete product information from the dietary supplement container was also recorded so that every reported dietary supplement could be matched or entered into a dietary supplements database. The NCHS maintains a dietary supplements database that contains product label information obtained from manufacturers of dietary supplements reported in NHANES; these data include the labeled dosage or serving size, ingredients, and the amounts of ingredients per serving. The average daily intake of nutrients from MVMS was calculated for individuals using the supplement consumption frequency and dosage. Nutrients from other dietary supplements not meeting the definition of MVMS were not included (e.g., single nutrient supplements, other multi-vitamin/multi-mineral dietary supplements with fewer than 9 nutrients). Supplement consumers were defined as those taking any amount of MVMS at any frequency. The frequency of MVMS usage was defined based on reported consumption during the past 30 days of: (1) 0 days (non-consumers of MVMS); (2) 1–10 days; (3) 11–20 days; and (4) ≥21 days. 

### 2.4. Biomarkers of Nutrient Deficiencies

As part of the NHANES in-person health examination in the Mobile Examination Center, participants provided a blood specimen for laboratory analyses. Values for serum levels of pyridoxal-5′-phosphate, cobalamin, ascorbic acid (vitamin C), 25-hydroxyvitamin D, transferrin receptor, and ferritin were obtained from laboratory files [22]. Body iron (mg/kg body weight) in females only was calculated as –[(log10((soluble transferrin receptor × 1000)/ferritin) − 2.8229)]/0.1207 where soluble transferrin receptor is in (mg/L) and ferritin is in (ng/mL) [23]. Because not all nutrient biomarkers were measured in each NHANES cycle, multiple cycles were combined to obtain an adequate sample size (for pyridoxal-5′-phosphate and body iron data from NHANES 2003–2010 were used; for serum cobalamin data from NHANES 2001–2012 were used; for ascorbic acid data from NHANES 2003–2006 were used; and for 25-hydroxyvitamin D data from NHANES 2001–2010 were used). The nutrient cut-off levels used to identify nutrient deficiency were: pyridoxal-5′-phosphate levels < 20 nmol/L for vitamin B_6_; serum cobalamin < 200 pg/mL for vitamin B_12_; serum ascorbic acid < 11.4 μmol/L for vitamin C; serum 25-hydroxyvitamin D < 30 nmol/L for vitamin D; and body iron < 0 mg/kg for iron deficiency [14]. 

### 2.5. Statistics

All statistical analyses were performed with SAS (version 9.2; SAS Institute Inc., Cary, NC, USA) and SUDAAN (version 11; Research Triangle Institute; Raleigh, NC, USA). NHANES survey weights, strata, and primary sampling units were used to calculate nationally representative estimates. Usual nutrient intakes (long-term intakes) from food only were estimated using two days of dietary intake in NHANES with the National Cancer Institute method [24] and with age groups, day of recall, weekday/weekend intake flag, and dietary supplement use (yes/no) flag as covariates. Nutrients provided by MVMS only were added to usual intake of food to obtain intakes from food and MVMS. The percentage of the population below the Estimated Average Requirement (EAR) using the cut-point method (except for iron where the probability method was used) for 17 micronutrients (calcium, copper, iron, magnesium, phosphorus, selenium, zinc, vitamin A, thiamin, riboflavin, niacin, folate, vitamin B_6_, vitamin B_12_, vitamin C, vitamin D, and vitamin E) and the percentage above the Tolerable Upper Intake Level (UL) for 12 micronutrients (calcium, copper, iron, phosphorus, selenium, zinc, vitamin A as retinol, folate as folic acid, vitamin B_6_, vitamin C, vitamin D, and vitamin E as added alpha-tocopherol) were assessed. A Z-statistic was used to test whether mean intakes and proportions of the population below the EAR or above the UL were similar between groups. Main comparisons were: (1) intakes from food alone and from food plus MVMS for MVMS consumers; and (2) intakes in non-consumers and the three groups of MVMS consumers, based on frequency of consumption. Logistic regression analyses were used to assess the association of MVMS usage on odds ratio of being below defined deficiency levels for certain nutrients in adults after adjustment for various covariates (age, gender, race/ethnicity, poverty income ratio, physical activity, and alcohol intake). To focus these analyses on MVMS, only subjects who consumed MVMS with no other dietary supplements were included in the logistic regression analyses. *p-*value < 0.01 was deemed statistically significant.

## 3. Results

### 3.1. MVMS Usage

About 28% of adults (*n* = 2623) reported taking a MVMS, including 3.6% (*n* = 349) for 1–10/30 days, 4.1% (*n* = 404) for 11–20/30 days, and 20.0% (*n* = 1870) for ≥21/30 days. 

### 3.2. Impact of MVMS Use and Frequency of Use

**Figure 1:**
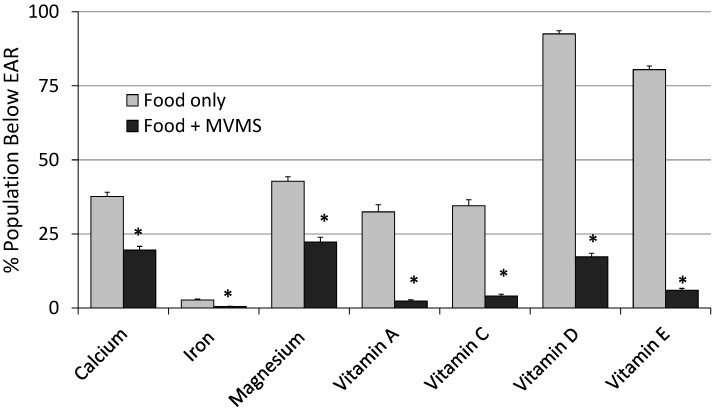
Prevalence of inadequacy (% of population below EAR) for “underconsumed nutrients” identified in 2015–2020 Dietary Guidelines for Americans from food only and food + MVMS among adults age 19 years and older reporting taking a MVMS. NHANES 2009–2012. * Significantly different from Food only at *p* < 0.01. EAR: estimated average requirement; MVMS: multi-vitamin/multi-mineral supplements; NHANES: National Health and Nutrition Examination Survey.

**Figure 2:**
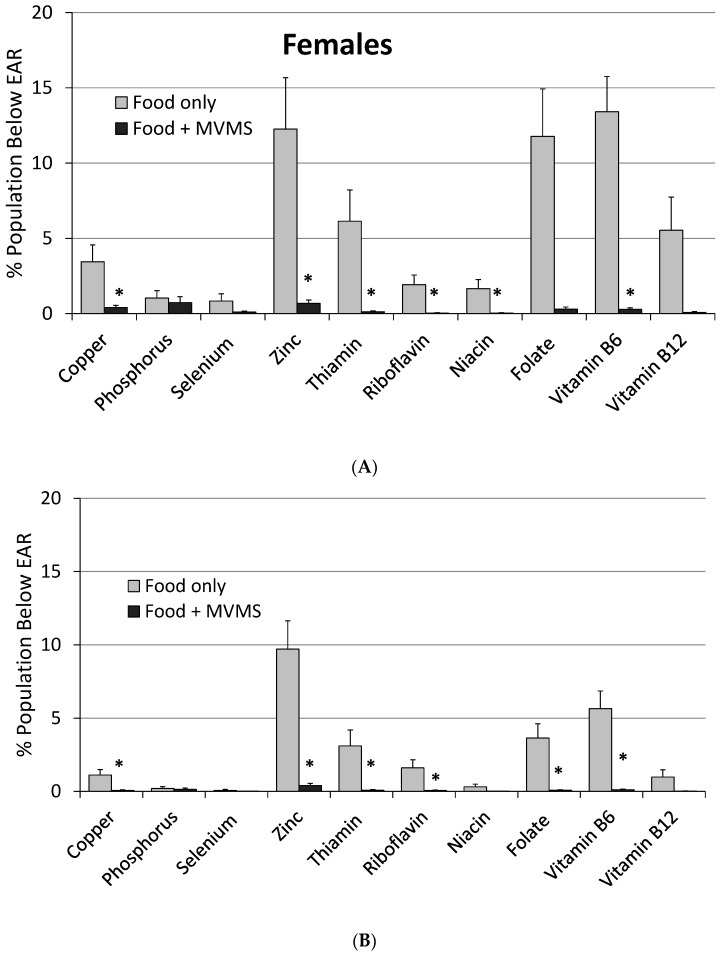
Prevalence of inadequacy (% of population below EAR) of intakes of other micronutrients from food only and food + MVMS among females and males age 19 years and older reporting taking a MVMS. NHANES 2009–2012. (**A**) Females; (**B**) Males. * Significantly different from Food only at *p* < 0.01. EAR: estimated average requirement; MVMS: multi-vitamin/multi-mineral supplements; NHANES: National Health and Nutrition Examination Survey.

Among adults who reported taking a MVMS (at any frequency), usual intake from food and MVMS combined was significantly higher (*p* < 0.01) for all micronutrients examined (except phosphorus) than from food only (Table 1). The prevalence of inadequacy (intakes < EAR) was significantly lower (*p* < 0.01) for seven of the DGA “underconsumed nutrients” and, except for calcium, magnesium, and vitamins D and E, was <5% for all 17 nutrients examined (Table 1 and Figure 1). MVMS consumers also had a higher (*p* < 0.01) prevalence of intakes above the UL for calcium, iron, selenium, zinc, vitamins A, B_6_, and folate (as folic acid) from food and MVMS compared to food only, however, all were ≤4% (Table 1). Similar results were obtained when the data were analyzed for male and female adults separately except that there was a slightly smaller difference in prevalence of inadequacy among male adults than among female adults (Figure 2). 

### 3.3. Impact of Frequency of MVMS Usage

**Figure 3:**
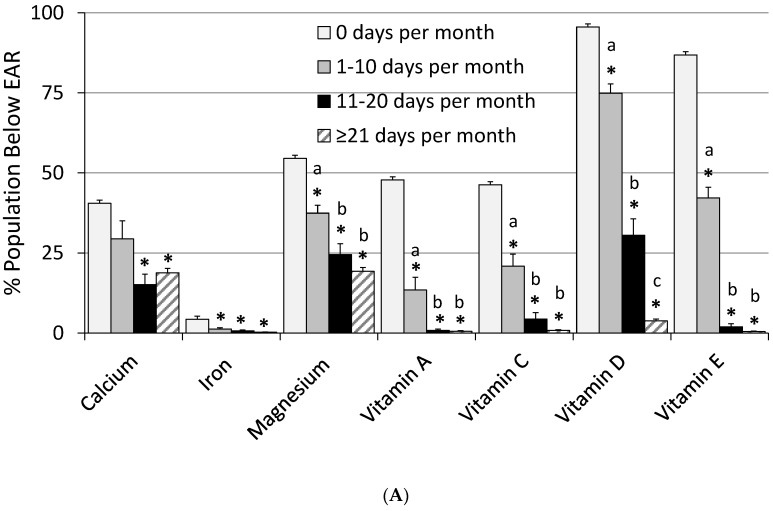

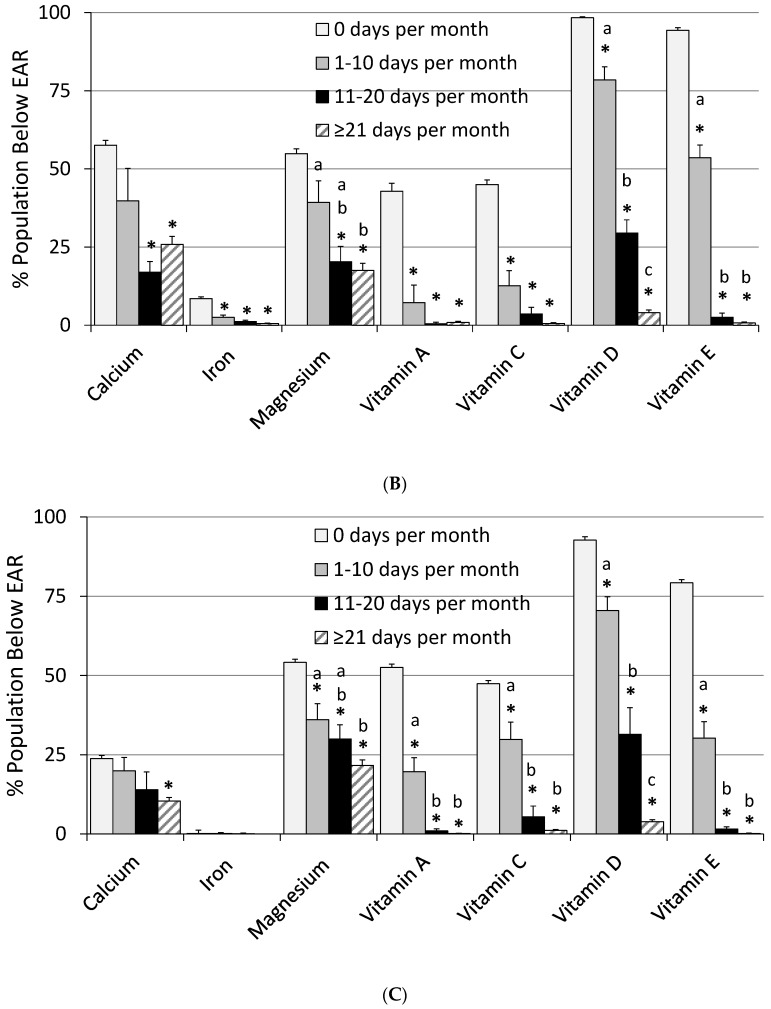
Prevalence of inadequacy (% of population below EAR) of “underconsumed nutrients” identified in 2015–2020 Dietary Guidelines for Americans for food + MVMS by frequency of MVMS intake among all adults (**A**); females (**B**); and males (**C**) age 19 years and older. NHANES 2009–2012. * Significantly different from 0 days per month at *p* < 0.01. ^a,b,c^ Values by frequency of MVMS with different superscripts are significantly different at *p* < 0.01. EAR: estimated average requirement; MVMS: multi-vitamin/multi-mineral supplements; NHANES: National Health and Nutrition Examination Survey.

**Figure 4:**
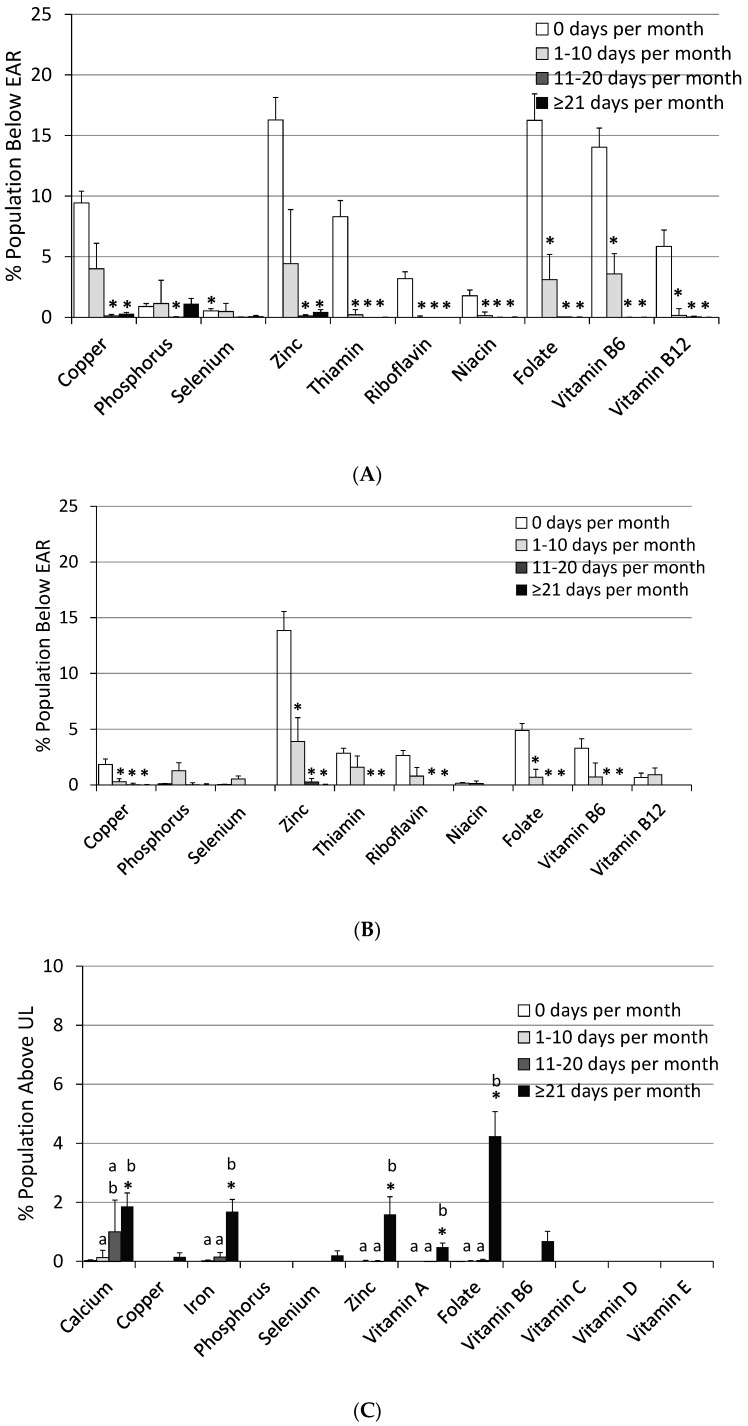

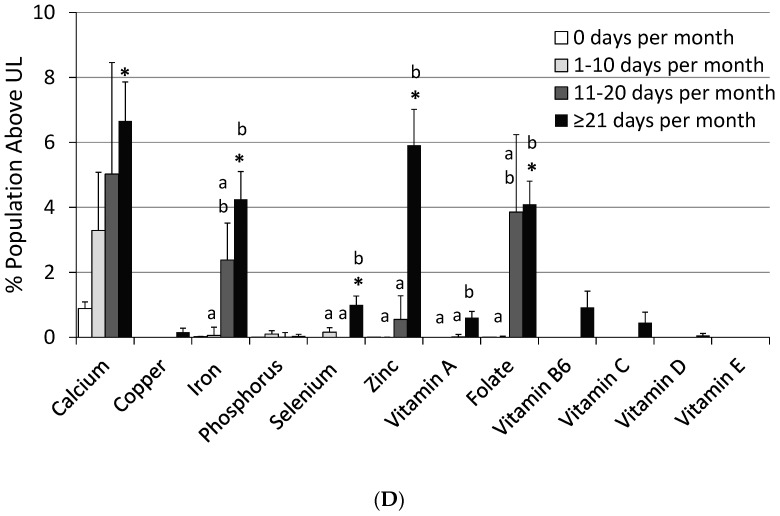
Prevalence of inadequacy (% of population below the EAR) and % population above the UL of other micronutrient intakes from food + MVMS by frequency of MVMS intake among adult females and males age 19 years and older. NHANES 2009–2012. (**A**) Females below EAR; (**B**) Males below EAR; (**C**) Females above UL; (**D**) Males above UL. * Significantly different from 0 days per month at *p* < 0.01. ^a,b,c^ Values by frequency of MVMS with different superscripts are significantly different at *p* < 0.01. EAR: estimated average requirement; MVMS: multi-vitamin/multi-mineral supplements; NHANES: National Health and Nutrition Examination Survey; UL: upper tolerable intake level.

The usual intakes for most micronutrients significantly increased (*p* < 0.01) with increased frequency of MVMS intake compared to not taking a MVMS (Table 2). Any frequency of MVMS usage was associated with significant reductions in inadequate intakes for all DGA “underconsumed nutrients” except for calcium with the lowest MVMS frequency (Figure 3). Prevalence of inadequacy for magnesium and vitamins A, C, D, and E also decreased significantly (*p* < 0.01) with increased frequency of MVMS intake. The prevalence of inadequacy for vitamins A, C, D, and E dropped to ≤4% of adults with MVMS intake at the highest frequency (≥21 days per 30 days). The percentage of the population above the UL, while extremely low for those not taking a MVMS, increased significantly for calcium, iron, selenium, zinc, vitamin A, vitamin B_6_, and folate for the highest frequency of MVMS usage, however, it was never more than ~4% of the population for any of the nutrients (Table 2). Again, similar results were observed when the data were analyzed for male and female adults separately. Reductions in inadequate intakes for all DGA “underconsumed nutrients” (Figure 3) and for percentages <EAR/>UL were similar except that there was a slightly larger impact among female adults for percentages <EAR than among male adults. Additionally, male adults had slightly higher percentages >UL than females (Figure 4). For many but not all nutrients, different MVMS usage frequencies were significantly different from one another in nutrient intakes and <EAR/>UL prevalence percentages (Table 2, Figure 3 and Figure 4). 

### 3.4. Impact of Frequency of MVMS Usage on Biomarkers of Nutrient Deficiencies

Those not taking a MVMS (0 days) had the following percentages of the population (SE) with biomarker-defined deficiency levels: 17.5 (0.7) for vitamin B_6_; 2.7 (0.2) for vitamin B_12_; 10.5 (1.1) for vitamin C; 8.5 (0.6) for vitamin D; and 7.3 (0.6) for total body iron (women only). The use of a MVMS at any frequency was associated with a significantly reduced risk (*p* < 0.01) of being at or below deficiency levels for the vitamins examined, but not body iron (Table 3). Compared to not using a MVMS, the most frequent MVMS usage (≥21 days per 30 days) was associated with a 58–76% reduced risk of being at or below deficiency levels for the vitamins examined (MVMS at any frequency was associated with a 58–69% lowered risk of nutrient deficiencies for vitamins B_6_, B_12_, C, and D). MVMS consumption was not associated with risk of low body iron stores (women only) (Table 3). Less frequent MVMS usage (11–20 days per 30 days) was also associated with significantly lower OR of being deficient for vitamin B_6_ and vitamin D compared to those not reporting use of MVMS.

## 4. Discussion

This report evaluated the impact of frequency of MVMS use on the prevalence of micronutrient inadequacies (defined as nutrient intakes below the EAR) and deficiencies (defined as having low levels of biomarkers of nutrient status) in the U.S. adult population. Similar to earlier reports [4,5,6], in the present analysis using NHANES data from 2009–2012, about 28% reported taking MVMS as defined in this study. Most MVMS consumers reported taking MVMS every day, with a smaller number of occasional or sporadic users. Use of a MVMS at any frequency significantly increased nutrient intakes and decreased the percent of the population with inadequate intakes for most micronutrients, especially “underconsumed nutrients”, as compared to food alone. More frequent MVMS use was associated with significantly higher nutrient intakes and lower prevalence of nutrient inadequacies, with these effects being more pronounced among regular users compared to sporadic users.

Micronutrient inadequacies are associated with adverse health effects such as neural tube defects, poor bone health (osteoporosis), impaired immune function, and impaired cognitive function, as well as chronic diseases, such as certain cancers, age-related eye diseases, hypertension, and possibly coronary heart disease and stroke [14,25,26]. Low intakes of calcium, potassium, iron (adolescent and adult females), dietary fiber, and vitamin D have been linked in the scientific literature to adverse health outcomes and hence are considered nutrients of public health concern [2]. The CDC’s Second National Report on Biochemical Indicators of Diet and Nutrition in the U.S. Population [14] reported that about 10% of the U.S. population had nutritional deficiencies (based on nutrient status markers), which could be as high as nearly one third for certain subpopulations. Micronutrient deficiencies are known to cause deficiency disease, such as rickets (vitamin D), scurvy (vitamin C), megaloblastic anemia (folate or vitamin B_12_), or iron-deficiency anemia. Our data indicate that use of MVMS at any frequency was associated with a 58–69% lowered risk of nutrient deficiencies for vitamins B_6_, B_12_, C, and D, and most frequent use of MVMS (≥21 days per 30 days) was associated with a 58–76% lowered risk. 

As expected, more frequent MVMS intake shifted the population to higher nutrient intakes and thus lowered the prevalence of nutrient inadequacies, but also increased the prevalence of intakes above the UL for several nutrients. However, even with the most frequent usage of MVMS, the prevalence of overconsumption was ≤4%. Further, about 45% or more of those exceeding the UL were within 10% of the UL (data not shown). However, this study only examined the nutrient contribution of MVMS and not other dietary supplements simultaneously consumed; 33.1 ± 1.3% of MVMS consumers were also taking a single-nutrient supplement (defined as a dietary supplement with one and only one nutrient at any level), and their use increased in MVMS consumers as frequency of MVMS consumption increased (20.8 ± 1.3%, 29.2 ± 2.5%, and 36.3 ± 1.5% in the 1–10, 11–21, and ≥21 days per 30 days groups, respectively). Concomitant consumption of MVMS and single supplements (and other non-MVMS supplements) needs to be more thoroughly evaluated as intakes may increase the percentage of the population exceeding the UL.

A major strength of our study was the use of a large, nationally representative, population-based sample of adults examining frequency of consumption of one common type of dietary supplement, MVMS. A limitation of our study was that the estimates relied on self-reported intakes, which require memory and are subject to bias, especially in those who are overweight or obese [27]. Additionally, self-reported dietary supplement intake was assumed to accurately reflect long-term MVMS intake patterns. Although the dietary supplement data were self-reported, about 80% of the time, NHANES interviewers saw the dietary supplement bottles/labels and verified the self-reporting accuracy. Furthermore, estimates of vitamins and minerals contributed by MVMS relied on the label declarations rather than analytic values. Only the impact of MVMS was evaluated, and not other types of dietary supplements concomitantly consumed, which would further increase intake. The results of this study should be interpreted with these limitations in mind.

## 5. Conclusions

Frequent use of MVMS is effective in increasing micronutrient intakes, decreasing prevalence of most nutrient inadequacies, and decreasing risk of deficiencies of vitamins B_6_, B_12_, C, and D in the U.S. adult population. Intake of MVMS also slightly increased the prevalence of consumption above the UL for calcium, iron, zinc, and folic acid, but was ≤4% for any nutrient.

## Figures and Tables

**Table 1:** Usual intake, prevalence of inadequacy (% of population below EAR) and % of population exceeding the UL of 17 micronutrients from food only and food + MVMS among adults age 19 years and older reporting taking a MVMS. NHANES 2009–2012.

Nutrient	Usual Intake		% Population Below EAR		% Population Above UL	
	Food Only	Food + Supplement.	Food Only	Food + Supplement	Food Only	Food + Supplement
Calcium (mg)	1061 ± 15	1277 ± 1 *	37.7 ± 1.4	19.6 ± 1.3 *	1.42 ± 0.29	3.66 ± 0.49 *
Copper (mg)	1.41 ± 0.02	2.40 ± 0.03 *	2.3 ± 0.6	0.22 ± 0.08 *	0.00 ± 0.00	0.11 ± 0.07
Iron (mg)	16.2 ± 0.2	23.8 ± 0.3 *	2.71 ± 0.33	0.50 ± 0.12 *	0.02 ± 0.01	2.25 ± 0.29 *
Magnesium (mg)	330 ± 4	389 ± 5 *	42.8 ± 1.5	22.3 ± 1.6 *	ND	ND
Phosphorus (mg)	1451 ± 14	1475 ± 18	0.58 ± 0.27	0.58 ± 0.27	0.01 ± 0.02	0.02 ± 0.02
Selenium (µg)	113 ± 1	177 ± 9 *	0.51 ± 0.23	0.06 ± 0.04	0.00 ± 000	0.35 ± 0.10 *
Zinc (mg)	12.1 ± 0.1	23.5 ± 0.3 *	11.0 ± 2.0	0.54 ± 0.1 *	0.00 ± 0.00	2.47 ± 0.41 *
Vitamin A (RAE, µg) ^a^	738 ± 20	1602 ± 26 *	32.5 ± 2.4	2.34 ± 0.43 *	0.00 ± 0.00	0.37 ± 0.09 *
Thiamin (mg)	1.71 ± 0.02	8.5 ± 1.3 *	4.74 ± 1.15	0.09 ± 0.03 *	--	--
Riboflavin (mg)	2.29 ± 0.03	7.5 ± 0.5 *	1.75 ± 0.54	0.04 ± 0.02 *	--	--
Niacin (mg)	26.1 ± 0.3	45.4 ± 0.7 *	0.98 ± 0.36	0.03 ± 0.01 *	ND	ND
Folate DFE (µg) ^b^	588 ± 9	1197 ± 14 *	7.96 ± 1.67	0.21 ± 0.07 *	0.00 ± 0.00	3.25± 0.36 *
Vitamin B_6_ (mg)	2.20 ± 0.03	8.72 ± 0.55 *	9.89 ± 1.44	0.19 ± 0.05 *	0.00 ± 0.00	0.57 ± 0.17 *
Vitamin B_12_ (µg)	5.55 ± 0.13	31.8 ± 2.4 *	3.33 ± 1.26	0.05 ± 0.02 *	--	--
Vitamin C (mg)	92.9 ± 2.6	188 ± 6 *	34.6 ± 2.0	4.09 ± 0.53 *	0.00 ± 0.00	0.15 ± 0.11
Vitamin D (µg)	5.17 ± 0.16	17.1 ± 0.3 *	92.5 ± 1.1	17.3 ± 1.2 *	0.00 ± 0.00	0.02 ± 0.02
Vitamin E (mg) ^c^	9.27 ± 0.16	37.3 ± 1.3 *	80.4 ± 1.2	5.96 ± 0.65 *	0.00 ± 0.00	0.00 ± 0.00

* Significantly different from Food Only column at *p* < 0.01; ND: Not determined as niacin and magnesium UL are based on a particular form/sources, which are not quantified in NHANES. ^a^ UL based on retinol; ^b^ UL based on folic acid; ^c^ UL based on added alpha-tocopherol. EAR: estimated average requirement; mg: milligram; MVMS: multi-vitamin/multi-mineral supplements; NHANES: National Health and Nutrition Examination Survey; RAE: retinol activity equivalents; DFE: dietary folate equivalents µg: microgram; UL: tolerable upper intake level.

**Table 2:** Usual intake, prevalence of inadequacy (% population below EAR), and % population exceeding tolerable upper intake level (UL) of 17 micronutrients from food + MVMS by frequency of MVMS intake among all adults age 19 years and older. NHANES 2009–2012.

Nutrients	Usual Intake				% Population Below EAR				% Population Above UL			
	0 Days Per Month	1 to 10 Days Per Month	11 to 20 Days Per Month	21 Days or More Per Month	0 Days Per Month	1 to 10 Days Per Month	11 to 20 Days Per Month	21 Days or More Per Month	0 Days Per Month	1 to 10 Days Per Month	11 to 20 Days Per Month	21 Days or More Per Month
Calcium (mg)	986 ± 9	1143 ± 57 *^,a^	1284 ± 47 *^,a,b^	1299 ± 21 *^,b^	40.5 ± 0.8	29.4 ± 5.6	15.2 ± 3.2 *	18.8 ± 1.4 *	0.47 ± 0.09	1.93 ± 0.85	2.89 ± 1.61	4.13 ± 0.62 *
Copper (mg)	1.30 ± 0.01	1.63 ± 0.03 *^,a^	2.11 ± 0.06 *^,b^	2.59 ± 0.04 *^,c^	5.66 ± 0.49	2.17 ± 1.1 *	0.11 ± 0.09 *	0.15 ± 0.06 *	0.00 ± 0.00	0.00 ± 0.00	0.00 ± 0.00	0.15 ± 0.10
Iron (mg)	15.3 ± 0.2	18.6 ± 0.5 *^,a^	22.9 ± 0.9 *^,b^	24.9 ± 0.5 *^,b^	4.3 ± 0.3	1.31 ± 0.40 *	0.67 ± 0.30 *	0.26 ± 0.11 *	0.01 ± 0.01	0.07 ± 0.13 ^a^	1.27 ± 0.56 ^a,b^	2.90 ± 0.46 *^,b^
Magnesium (mg)	304 ± 3	345 ± 6 *^,a^	369 ± 15 *^,a,b^	401 ± 6 *^,b^	54.6 ± 1.2	37.4 ± 2.5 *^,a^	24.5 ± 3.4 *^,b^	19.3 ± 1.2 *^,b^	ND	ND	ND	ND
Phosphorus (mg)	1409 ± 9	1468 ± 38	1506 ± 54	1467 ± 26	0.49 ± 0.13	1.15 ± 0.91	0.06 ± 0.08 *	0.58 ± 0.23	0.00 ± 0.00	0.04 ± 0.05	0.00 ± 0.06	0.01 ± 0.03
Selenium (µg)	115 ± 1	126 ± 4 *^,a^	152 ± 6 *^,b^	191 ± 12 *^,c^	0.28 ± 0.11	0.44 ± 0.42 *	0.00 ± 0.02	0.04 ± 0.03	0.00 ± 0.00	0.08 ± 0.07 ^a^	0.00 ± 0.00 ^a^	0.51 ± 0.14 *^,b^
Zinc (mg)	11.7 ± 0.1	14.4 ± 0.4 *^,a^	19.8 ± 0.5 *^,b^	25.8 ± 0.3 *^,c^	15.1 ± 1.4	3.87 ± 2.40 *	0.17 ± 0.10 *	0.22 ± 0.08 *	0.00 ± 0.00	0.00 ± 0.01 ^a^	0.27 ± 0.32 ^a^	3.59 ± 0.53 *^,b^
Vitamin A (µg) ^a^	620 ± 14	894 ± 38 *^,a^	1261 ± 48 *^,b^	1797 ± 38 *^,c^	47.8 ± 2.2	13.4 ± 4.0 *^,a^	0.81 ± 0.46 *^,b^	0.57 ± 0.20 *^,b^	0.00 ± 0.00	0.00 ± 0.00 ^a^	0.00 ± 0.04 ^a^	0.53 ± 0.11 *^,b^
Thiamin (mg)	1.64 ± 0.01	3.28 ± 0.22 *^,a^	4.92 ± 0.47 *^,b^	10.2 ± 1.5 *^,c^	5.51 ± 0.62	0.89 ± 0.68 *	0.00 ± 0.00 *	0.00 ± 0.00 *	--	--	--	--
Riboflavin (mg)	2.13 ± 0.03	3.68 ± 0.22 *^,a^	5.40 ± 0.45 *^,b^	8.59 ± 0.46 *^,c^	2.90 ± 0.37	0.53 ± 0.37 *	0.00 ± 0.00 *	0.00 ± 0.00 *	--	--	--	--
Niacin (mg)	26.2 ± 0.2	30.1 ± 1.1 *^,a^	39.3 ± 0.9 *^,b^	49.5 ± 0.9 *^,c^	0.95 ± 0.23	0.08 ± 0.22 *	0.00 ± 0.01 *	0.00 ± 0.00 *	ND	ND	ND	ND
Folate DFE (µg) ^b^	551 ± 7	728 ± 37 *^,a^	1028 ± 33 *^,b^	1318 ± 16 *^,c^	10.5 ± 1.2	2.19 ± 1.12 *	0.00 ± 0.01 *	0.01 ± 0.005 *	0.00 ± 0.00	0.01 ± 0.02 ^a^	1.58 ± 1.10 ^a,b^	4.05 ± 0.58 *^,b^
Vitamin B_6_ (mg)	2.13 ± 0.03	3.71 ± 0.23 *^,a^	5.87 ± 0.50 *^,b^	10.2 ± 0.5 *^,c^	8.56 ± 0.79	2.01 ± 0.86 *	0.00 ± 0.00 *	0.00 ± 0.00 *	0.00 ± 0.00	0.00 ± 0.00 ^a^	0.00 ± 0.00 ^a^	0.79 ± 024 *^,b^
Vitamin B_12_ (µg)	5.3 ± 0.1	8.9 ± 0.5 *^,a^	16.4 ± 1.0 *^,b^	39.0 ± 2.3 *^,c^	3.31 ± 0.61	0.38 ± 0.46 *	0.04 ± 0.04 *	0.00 ± 0.00 *	--	--	--	--
Vitamin C (mg)	83.9 ± 2.6	110 ± 7 *^,a^	158 ± 9 *^,b^	208 ± 8 *^,c^	46.3 ± 1.4	20.9 ± 3.8 *^,a^	4.37 ± 2.02 *^,b^	0.83 ± 0.23 *^,b^	0.00 ± 0.00	0.00 ± 0.00	0.00 ± 0.00	0.21 ± 0.15
Vitamin D (µg)	4.8 ± 0.1	8.0 ± 0.04 *^,a^	13.2 ± 0.6 *^,b^	19.5 ± 0.3 *^,c^	95.6 ± 0.6	74.9 ± 2.9 *^,a^	30.5 ± 5.1 *^,b^	3.86 ± 0.50 *^,c^	0.00 ± 0.00	0.00 ± 0.00	0.00 ± 0.00	0.03 ± 0.03
Vitamin E (mg) ^c^	8.3 ± 0.1	13.8 ± 0.4 *^,a^	25.9 ± 1.3 *^,b^	43.8 ± 1.1 *^,c^	86.9 ± 1.0	42.2 ± 3.3 *^,a^	2.01 ± 0.90 *^,b^	0.50 ± 0.16 *^,b^	0.00 ± 0.00	0.00 ± 0.00	0.00 ± 0.00	0.00 ± 0.00

* Significantly different from 0 days column at *p* < 0.01; MVMS: multi-vitamin/multi-mineral supplements; ^a,b,c^ Values by frequency of MVMS with different superscripts are significantly different at *p* < 0.01. ND: Not determined as niacin and magnesium UL are based on a particular form/sources, which are not quantified in NHANES. ^a^ UL based on retinol; ^b^ UL based on folic acid; ^c^ UL based on added alpha-tocopherol. EAR: estimated average requirement; mg: milligram; MVMS: multi-vitamin/multi-mineral supplements; NHANES: National Health and Nutrition Examination Survey; µg: microgram; UL: tolerable upper intake level.

**Table 3:** Impact of MVMS intake frequency on odds ratios of risk of being below defined deficiency level for certain nutrients in adults.

Variables	N	Odds Ratio (99% CI) of Deficiency by Frequency of MVMS Use				
		0 Days	1 to 30 Days	1 to 10 Days	11 to 20 Days	21 Days or More
Deficient Vitamin B_6_ (<20 nmol/L) ^a^	14,596	1.00	0.36 (0.27, 0.48) *	0.47 (0.27, 0.82) *	0.33 (0.19, 0.56) *	0.34 (0.22, 0.52) *
Deficient Vitamin B_12_ (<200 pg/mL) ^b^	13,345	1.00	0.42 (0.22, 0.80) *	0.43 (0.12, 1.57)	0.42 (0.04, 4.60)	0.42 (0.22, 0.78) *
Deficient Vitamin C (<11.4 µmol/L) ^c^	6423	1.00	0.31 (0.12, 0.82) *	0.34 (0.09, 1.37)	0.44 (0.08, 2.30)	0.27 (0.09, 0.84) *
Deficient Vitamin D (<30 mmol/L) ^d^	17,655	1.00	0.34 (0.23, 0.48) *	0.88 (0.40, 1.95) ^a^	0.22 (0.08, 0.59) *^,b^	0.24 (0.15, 0.40) *^,b^
Deficient Body Iron (<0 mg/kg) ^a^	3916	1.00	0.63 (0.33, 1.22)	0.58 (0.16, 2.14)	1.10 (0.40, 3.07)	0.50 (0.18, 1.39)

* Significantly different from 0 Days at *p* < 0.01. ^a,b,c^ Values by frequency of MVMS with different superscripts are significantly different at *p* < 0.01. Those not taking an MVMS (0 days) had the following percentages of the population (SE) with deficiency levels of vitamin B_6_ (<20 nmol/L), vitamin B_12_ (<200 pg/mL)_,_ vitamin C (<11.4 μmol/L), vitamin D (<30 nmol/L), and body iron (<0 mg/kg; women only):, 17.5 (0.7), 2.7 (0.2), 10.5 (1.1), 8.5 (0.6) and 7.3 (0.6)%, respectively. ^a^ NHANES 2003–2010; ^b^ NHANES 2001–2012; ^c^ NHANES 2003–2006; ^d^ NHANES 2001–2010. CI: confidence interval; kg: kilogram; L: liter; mg: milligrams; mL: milliliter; mmol: millimoles; MVMS: multi-vitamin/multi-mineral supplements; NHANES: National Health and Nutrition Examination Survey; nmol: nanomoles; pg: picograms; µmol: micromoles.
